# A Comparison of Preschoolers’ Physical Activity Indoors versus Outdoors at Child Care

**DOI:** 10.3390/ijerph15112463

**Published:** 2018-11-05

**Authors:** Pooja S. Tandon, Brian E. Saelens, Chuan Zhou, Dimitri A. Christakis

**Affiliations:** 1 Center for Child Health, Behavior, and Development, Seattle Children’s Research Institute, Seattle, WA 98145, USA; brian.saelens@seattlechildrens.org (B.E.S.); chuan.zhou@seattlechildrens.org (C.Z.); dimitri.christakis@seattlechildrens.org (D.A.C.); 2 Department of Pediatrics, University of Washington, Seattle, WA 98195, USA

**Keywords:** childhood obesity, outdoor play, daycare, GPS, sedentary behavior

## Abstract

The aims of this study were to quantify and examine differences in preschoolers’ indoor and outdoor sedentary time and physical activity intensity at child care using GPS devices and accelerometers. We conducted an observational study of 46 children (mean age 4.5 years, 30 boys, 16 girls) from five child care centers who wore accelerometers and GPS devices around their waists for five days during regular child care hours. GPS signal-to-noise ratios were used to determine indoor vs. outdoor location. Accelerometer data were categorized by activity intensity. Children spent, on average, 24% of child care time outdoors (range 12–37% by site), averaging 74 min daily outdoors (range 30–119 min), with 54% of children spending ≥60 min/day outdoors. Mean accelerometer activity counts were more than twice as high outdoors compared to indoors (345 (95) vs. 159 (38), (*p* < 0.001)), for girls and boys. Children were significantly less sedentary (51% of time vs. 75%) and engaging in more light (18% vs. 13%) and moderate-to-vigorous (MVPA) (31% vs. 12%) activity when outdoors compared to indoors (*p* < 0.001). To achieve a minute of MVPA, a preschooler needed to spend 9.1 min indoors vs. 3.8 min outdoors. Every additional 10 min outdoors each day was associated with a 2.9 min increase in MVPA (2.7 min for girls, 3.0 min for boys). Preschool-age children are twice as active and less sedentary when outdoors compared to indoors in child care settings. To help preschoolers achieve MVPA recommendations and likely attain other benefits, one strategy is to increase the amount of time they spend outdoors and further study how best to structure it.

## 1. Introduction

Physical activity (PA) lowers the risk of childhood obesity and is independently associated with numerous other immediate and long-term health benefits, including the promotion of cardiovascular, musculoskeletal, and mental health [1,2]. The amount of time preschool-age children spend outdoors is thought to correlate with their PA levels, although limitations exist in the way outdoor activity has been measured [3,4,5]. Most prior studies have relied on potentially biased child/parent reports or resource intensive direct observation for quantification of children’s outdoor time and/or physical activity [5,6,7,8]. Greater precision is needed in measuring location based physical activity to better tailor interventions and estimate their effects. More feasible and objective measures for determining indoor vs. outdoor location are now available in small, wearable global positioning system (GPS) devices, and algorithms have been developed to integrate GPS data with PA data from accelerometers [9]. However, estimates of preschoolers’ outdoor time and related activity levels outdoors using these more objective measures are not yet available.

The majority of 3- to 5-year-olds in the US spend over 30 h/week in non-parental child care [10]. Best practice guidelines for child care recommend two to three daily occasions of active outdoor play, totaling 60 to 90 min for preschoolers [11]. These guidelines also recommend at least 60 to 120 min per day of PA for preschool-age children, which nearly half are not attaining [12,13]. Understanding and documenting differences in preschoolers’ activity levels indoors versus outdoors has the potential to influence policy and encourage practices which promote physical activity in child care settings. Previous research has also shown that there is a gender disparity in physical activity levels, with boys more active than girls from a young age [14,15,16,17], but it is not known if that may be related to gender differences in indoor vs. outdoor activity. The primary aims of this preliminary study were to quantify and examine the differences in preschoolers’ indoor and outdoor sedentary time and physical activity intensity at child care using GPS devices and accelerometers.

## 2. Materials and Methods

### 2.1. Participants and Procedures

Preschool-age children (3- to 5-years-old) were recruited from a convenience sample of 5 child care centers located in the metropolitan Seattle area, which included a hospital-affiliated child care center, a Head Start program, and 3 private preschools. One classroom at each center was chosen for participation by the center director, and teachers were asked to maintain their usual daily schedules. If the parent(s) provided written informed consent, the child was asked for verbal assent. The child wore an Actigraph GT3X+ device on the right hip and a QStarz GPS device on the left hip while at child care, with a goal of getting 5 days of data on each child. The ActiGraph has been validated and calibrated for use among preschool children [18]. The QStarz device has also been previously validated in preschoolers to determine indoor vs. outdoor time [9]. Research staff placed the devices on children when they arrived at the child care center in the morning and removed them when children left each day. Additional information regarding teacher-child ratios and presence/use of indoor and outdoor play spaces was collected by research staff through inquiry and observation. Data were collected in fall/winter 2011 and weather conditions (“sunny”, “partly cloudy”, and “overcast”) on data collection days were noted. Study procedures were approved by the Seattle Children’s Hospital IRB.

### 2.2. Data Collection and Processing

GPS devices were initialized using QTravel software (QStarz International, Taipei, Taiwan) and set to record data at 15-s epochs. The Actigraph GT3X+ accelerometers were initialized using ActiLife (Actigraph, Pensacola, FL, USA) after the computer clock was synchronized to UTC time used by the GPS and data were later aggregated to 15-s epochs. Per research on the reliability of accelerometer data in preschoolers, a minimum of 3 h of wear time was required to be included in analysis, especially since some programs were half-day only [19].

GPS and accelerometer data were merged by day/time stamp using the Personal Activity and Location Measurement System (PALMS), which is a web-based software platform to merge and process data from different devices [20]. After spurious GPS data points are filtered using standard techniques, specific algorithms which utilize satellite signal strength or signal-to-noise (SNR) ratios detected by GPS devices were used to distinguish indoors from outdoors. When the satellite SNR value is greater than a set parameter (250), the fix is classified as outdoor. Direct observation was conducted for 2 of the 5 days at each center, and this information was used to validate the GPS data regarding indoors versus outdoor location with high reliability (sensitivity: 82%, specificity: 88%) [9].

### 2.3. Analysis

Data were analyzed using STATA (StataCorp. 2011. Stata Statistical Software: Release 12. College Station, TX, USA). We aggregated epoch level data to generate daily duration of time spent at different activity levels indoors and outdoors for each child and subsequently used child-level data as the unit of analysis. Accelerometer activity counts were categorized into sedentary, light, or moderate-to-vigorous (MVPA) activity using Pate [18], criteria as these have been calibrated and validated using metabolic criterion (measured oxygen consumption/indirect calorimetry). These results are reported using percentage of time in each activity intensity level to make comparison easier, as there were different total wear times/denominators. We also analyzed data using actual activity counts and minutes in each activity intensity level, which were compared between indoors and outdoors using child paired *t*-tests. We then ran a linear mixed effects model (with three nested levels: day nested within child, nested within center) to estimate the association between outdoor MVPA and total outdoor time.

## 3. Results

Forty-six children (mean age 4.5 years, 64% boys) from five centers participated. Characteristics of the children and child care centers are presented in Table 1. Children spent 24% of their time at child care outdoors, which varied by site (12%, 19%, 37%, 17%, 35% for sites 1–5, respectively). The site with the lowest proportion of outdoor time was the only center that did not have an outdoor playground. The two centers where children spent more than a third of their time in this study outdoors reported not having an indoor play area large enough for activities such as running. Three centers had children moving between indoors and outdoors as a group, while two centers allowed discretion such that children could choose, at certain times of the day, if they wanted to play outdoors. Across all centers, children were outdoors for an average of 74 min daily, ranging from 30–119 min. Results by child care are presented in Table 2. Fifty-four percent of children spent an average of at least 60 min per day outdoors. When examined by gender, 66% of boys and 35% of girls spent, on average, at least 60 min per day outdoors. The median outdoor time was 39.3 min for girls and 70.4 min for boys. In the adjusted analysis, we did not find significant differences in daily MVPA minutes and percent of daily MVPA across centers.

Children had mean accelerometer activity counts which were significantly higher when outdoors compared to indoors (345 (95) vs. 159 (38), (*p* < 0.001)) for both girls (339.8 (115.0) vs. 160.2 (42.3), (*p* < 0.001)) and boys (353.0 (78.8) vs. 160.0 (35.4), (*p* < 0.001)). Children were significantly less sedentary (51% of time vs. 75%) and engaging in more light (18% vs. 13%) and moderate-to-vigorous (MVPA) (31% vs. 12%) activity when outdoors compared to indoors (*p* < 0.001). Average amount of MVPA attained indoors was 25.91 (10.34) min vs. 22.97 (15.35) outdoors. One additional minute of MVPA, was correlated with 9.1 min indoors vs. 3.8 min outdoors. Thirty percent (14 children) achieved an average of at least 60 min of daily MVPA, with no difference by gender. Overall, every additional 10 min outdoors each day was associated with a 2.9 min increase in MVPA (2.7 min for girls, 3.0 min for boys). When controlling for total duration, sex, and weather, being outdoors was associated with 23.6 min more MVPA time. Child sex and weather were not significant predictors of MVPA minutes.

## 4. Discussion

This study provides objectively measured evidence that preschool-age children are more active and less sedentary when outdoors; in fact, they are twice as active outdoors versus indoors. Yet, only half the children in this study spent the recommended ≥60 min of their child care day outdoors [11]. Less than 1/3 of children achieved ≥60 min/day of MVPA.

Our results are consistent with previous studies that suggest children in US child care settings are sedentary for most of their attendance time and not meeting physical activity recommendations [12,21]. While some previous studies have found an association with parent-reported time spent outdoors and physical activity [22,23], one study found that relationship to be true for 5 to 6-year-olds but not 3 to 4-year-olds [24]. Perhaps our study can more accurately examine the relationship between outdoor time and physical activity given our use of GPS devices rather than parent report. Our results suggest that one strategy to increase preschoolers’ overall PA more efficiently, and likely attain other benefits, might be to increase the amount of time they spend outdoors during child care hours. A study which compared indoor and outdoor physical activity in preschoolers in Swedish and US settings found that more MVPA was observed outdoors vs. indoors in both countries, but US preschoolers spent a significantly lower proportion of child care attendance time outdoors (18%) than children in Sweden (46%) [25].

However, it may not be sufficient to just increase the number of minutes children are outdoors. Based on our study, children would need to be outdoors for 3.4 h to attain the recommended 60 min of MVPA, which may not be feasible in many settings and there may be a ceiling effect. One intervention study did test whether increasing 17 preschoolers’ outdoor recess time from 60 to 120 min for 2 days would increase physical activity, and the researchers found no change [26]. However, the sample size of this study was small and the intervention period brief. Another study found that structured outdoor playtime increased preschoolers’ PA levels more than free play [27], suggesting that other modifications besides just increasing the outdoor time may be needed such as improving the outdoor environment, conducting teacher training, and scheduling more frequent, shorter intervals of outdoor play. A recent randomized controlled trial found that scheduling multiple shorter periods of outdoor free play compared to a single longer period (with the same total duration of outdoor time) significantly increased children’s time in MVPA at child care by about 5 min [28]. Another study found that shorter, more frequent outdoor play periods did significantly increase MVPA in preschoolers, but this was not sustained at 6 or 12 months’ follow up [29].

In our sample, outdoor play opportunities differed between preschool sites. Whereas this study did not include enough sites to examine the relationship between children’s outdoor time and child care environments, this highlights room for improvement at some sites for more outdoor play opportunities and how they are structured. In the present study, Site 4 and, to some extent, Site 5 allowed children flexibility and choice in being outdoors vs. indoors during certain times of the day. This is reflected in the larger variability by child in outdoor time and is also driving the gender difference in outdoor time. While girls spent, on average, a smaller proportion of their time outdoors, there was, interestingly, no difference in the percentage of girls achieving 60 min of MVPA.

Also interesting was the finding that the centers without large indoor play areas had the greatest proportion of time spent outdoors, suggesting perhaps that the presence of an indoor play area may be perceived as an adequate alternative to outdoor play. In our results, however, the centers with indoor play areas did not have children attaining higher activity intensity indoors.

There are some study limitations. First, the sample of children was small (and with more boys), so larger samples with diversity in geographic and child care center characteristics are needed to determine generalizability. Second, child care centers in this study were not in highly urban areas where high rise buildings may distort satellite signals, so the validity of indoor/outdoor location based on GPS needs verification in other settings. Third, while limited weather information was collected, not all sites experienced the range of weather possibilities during the study days. It will be important to examine weather/seasonal and other factors (e.g., policies about going outside) that may be impacting preschool children’s outdoor time in child care. Finally, research that focuses on the entire day would provide a more holistic view of 24-h movement behaviors of children, which are increasingly recognized as important for health.

## 5. Conclusions

The current findings, based on objective measurements, reinforce the idea that preschoolers are over twice as active and less sedentary outdoors. Therefore, it behooves policy makers, health care providers, and others who have the power to influence how and where children spend time in child care settings, to promote and support more outdoor time. Future work in diverse and larger populations that helps elucidate how best to increase outdoor time and how it should be scheduled and structured to best facilitate active play will be an important next step.

## Figures and Tables

**Table 1:** Descriptive characteristics of children and preschool programs.

	Preschool 1	Preschool 2	Preschool 3	Preschool 4	Preschool 5
Number of study participants	6	11	7	12	10
Average age of participants, years (SD)	3.9 (0.5)	5.0 (0.1)	4.2 (0.6)	4.3 (0.7)	4.6 (0.30
Participants Sex, *N* (%)					
Female	3 (50%)	5 (45%)	2 (29%)	4 (33%)	2 (20%)
Male	3 (50%)	6 (55%)	5 (71%)	8 (67%)	8 (80%)
Child:Teacher ratio in preschool classrooms	7:1	9:1	9.5 or 6.3:1	10:1	5:1
Average # of days accelerometer worn (SD)	5.5 (0.5)	5.2 (1.1)	4.1 (0.7)	3.9 (1.2)	4.6 (0.5)
Average accelerometer wear, min/day (SD)	226 (11)	233 (4)	310 (19)	324 (61)	317 (45)
Outdoor playground on site? (Y/N)	N	Y	Y	Y	Y
Indoor play area sufficient for all activities, including running (by director report)? (Y/N)	Y	Y	N	Y	N
Child discretion allowed in choosing outdoors or indoors? (Y/N)	N	N	N	Y	Y-occasionally

**Table 2:** Physical activity by indoor and outdoor location.

Preschool Site	Daily Minutes Mean (SD)		Activity Counts/min Mean (SD)		Sedentary % Mean (SD)		Light PA % Mean (SD)		MVPA % Mean (SD)	
	Indoors	Outdoors	Indoors	Outdoors	Indoors	Outdoors	Indoors	Outdoors	Indoors	Outdoors
1	198.9 (10.5)	29.9 (3.3)	155.9 (39.5)	262.6 (79.8)	75.4 (6.4)	61.0 (10.0)	12.7 (2.9)	18.5 (6.2)	11.9 (3.5)	20.6 (5.0)
2	189.0 (14.0)	46.4 (10.3)	152.6 (31.9)	367.6 (70.2)	77.3 (4.8)	45.3 (8.7)	11.1 (2.3)	20.0 (5.0)	11.5 (2.8)	34.7 (7.9)
3	196.2 (27.1)	119.2 (14.3)	141.0 (37.0)	396.8 (120.1)	76.9 (5.1)	48.8 (8.1)	12.4 (2.1)	19.3 (1.4)	10.7 (3.6)	32.0 (8.1)
4	259.7 (82.3)	62.2 (39.9)	166.0 (42.8)	361.1 (89.9)	73.5 (6.6)	45.4 (11.1)	13.5 (3.0)	20.2 (2.5)	12.9 (3.9)	34.4 (9.4)
5	204.6 (27.5)	113.1 (30.5)	170.3 (41.4)	311.6 (87.6)	72.6 (6.9)	54.9 (10.0)	14.4 (3.7)	16.5 (2.4)	13.0 (3.8)	28.6 (9.5)
Overall	213.2 (52.6)	73.8 (42.2)	158.6 (38.4)	344.5 (94.9)	75.0 (6.1)	50.0 (10.9)	12.9 (3.0)	19.0 (3.9)	12.1 (3.5)	31.0 (9.3)
Difference indoors vs. outdoors, Mean (95% CI)	139.4 (116.7, 162.1) * d = 1.8		−185.9 (−211.2, −160.5) * d = −2.2		25.0 (22.0, 28.0) * d = 2.4		−6.1 (−7.4, −4.8) * d = −1.4		−18.9 (−21.4, −16.4) * d = −2.3	

* *p* < 0.001 (indoor vs outdoor, based on paired *t*-tests).
